# Supplementary material for: Electrochemical deposition of N-heterocyclic carbene monolayers on metal surfaces
Source: Nat Commun. 2020 Nov 11;11:5714. doi: 10.1038/s41467-020-19500-7 (PMC7658200; doi:10.1038/s41467-020-19500-7)
Supplement: Supplementary file 1 — Supplementary Information [file 41467_2020_19500_MOESM1_ESM.pdf]

Supplementary Information

Electrochemical Deposition of N-Heterocyclic Carbene Monolayers on Metal Surfaces

Einav Amit[a,b+], Linoy Dery[a,b+], Shahar Dery[a,b], Suhong Kim[c], Anirban Roy[d], Qichi Hu[d], Vitaly Gutkin[b], Helen Eisenberg[a,e], Tamar Stein[a,e], Daniel Mandler[a,b], F. Dean Toste[c] and Elad Gross[*] [a,b]

[a] *Institute of Chemistry, The Hebrew University, Jerusalem 91904, Israel*

[b] *The Center for Nanoscience and Nanotechnology, The Hebrew University, Jerusalem 91904, Israel*

[c] *Department of Chemistry, University of California, Berkeley, CA 94720, USA*

[d] *Bruker Nano Surfaces Division, 112 Robin Hill Road, Santa Barbara, CA 93117, USA*

[e] *The Fritz Haber Center for Molecular Dynamics Research, The Hebrew University, Jerusalem 91904, Israel*

Supplementary Figures

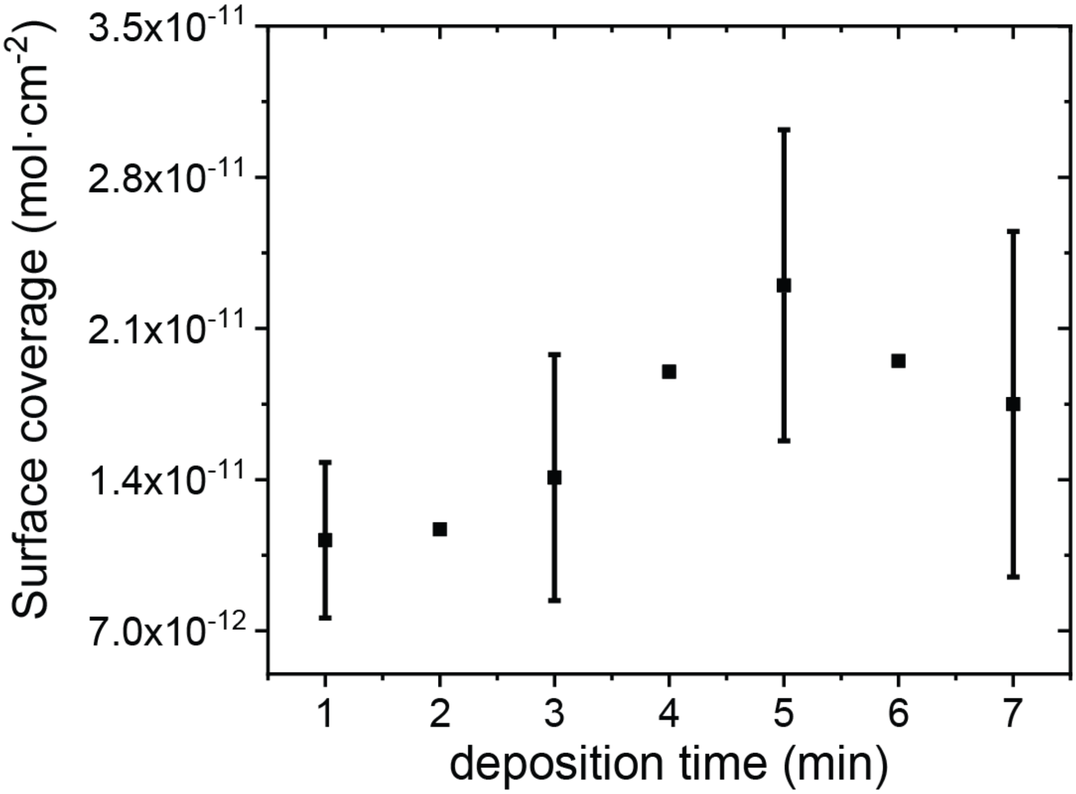

Supplementary Figure 1: The influence of electrodeposition duration on the surface density of NO_2-NHCs as quantified by electroreduction measurements. Error bars represent the SD values of at least 3 different samples.

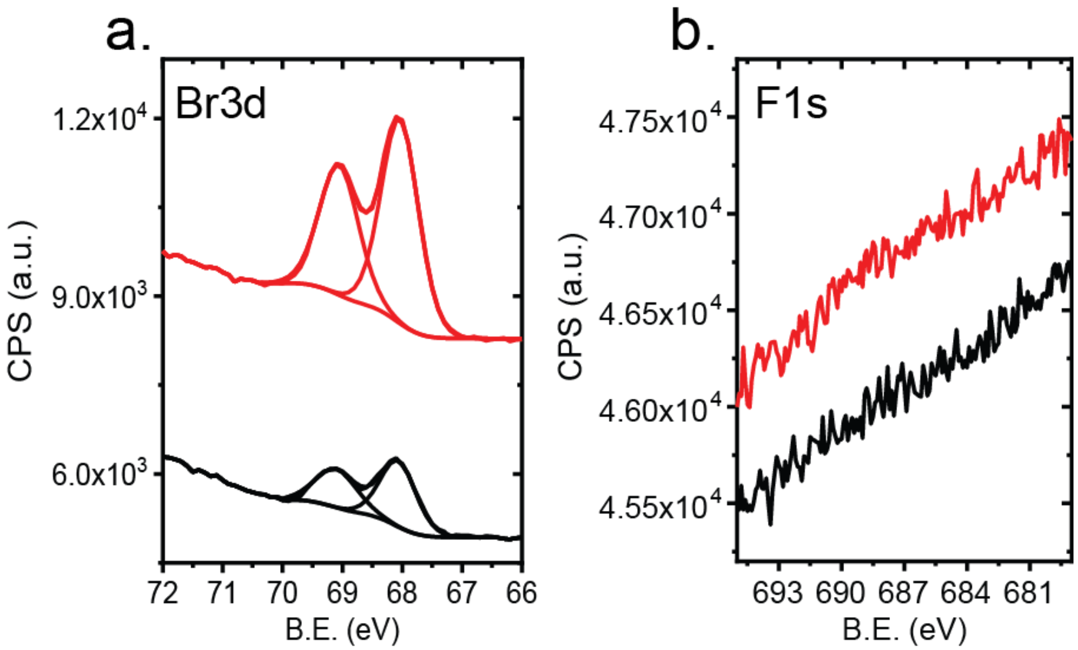

Supplementary Figure 2: Br3*d* (**a**) and F1*s* (**b**) XPS signals from Au surface on which NO_2-NHCs were EC-deposited (black-colored spectra) and deposited by base-induced deprotonation (red colored spectra).

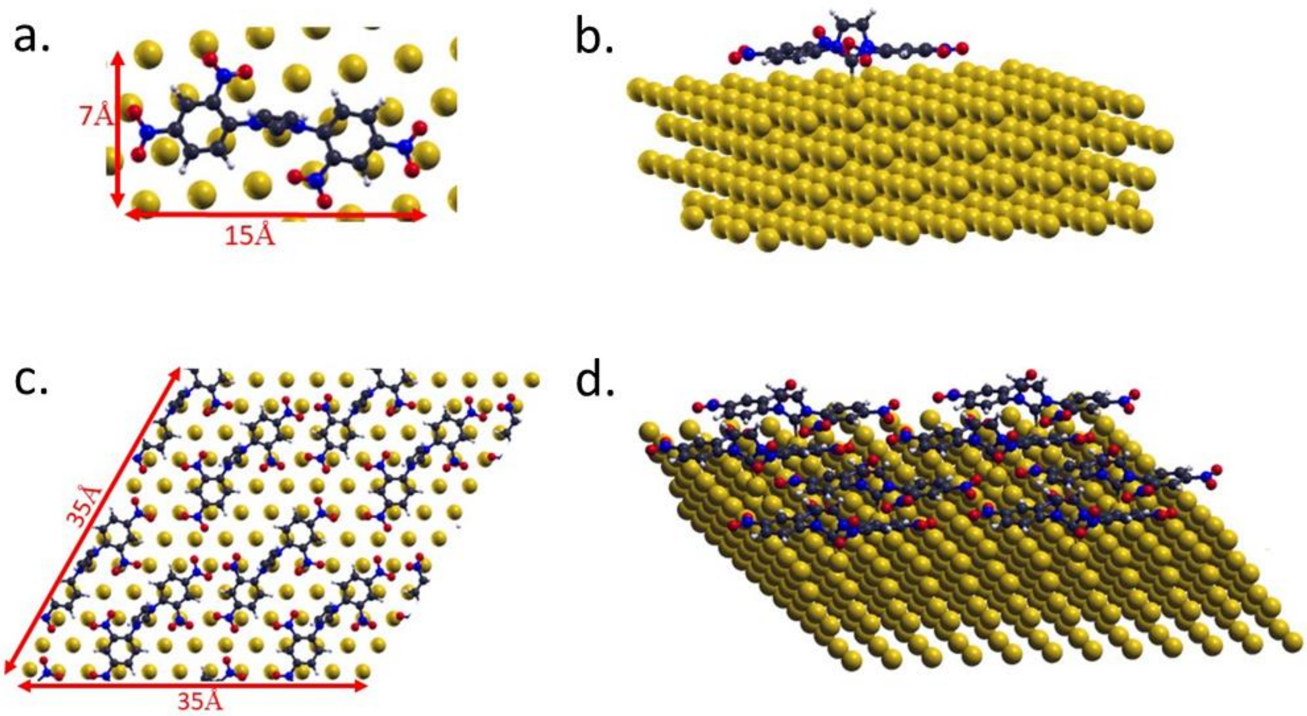

Supplementary Figure 3: DFT calculation of the optimized adsorption geometry of single (**a-b**) and closely packed (**c-d**) NO_2-NHC molecules on Au (111) surface.

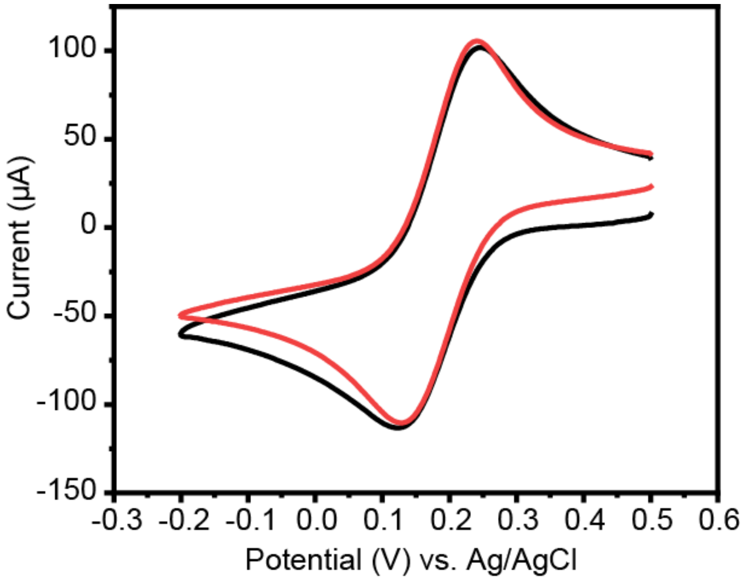

Supplementary Figure 4: CVs of 2 mM $[Fe(CN)_6]^{3-}$ in 0.1 M KCl recorded with Au electrode before and following EC-deposition of NO_2-NHCs (black- and red-colored voltammogram, respectively).

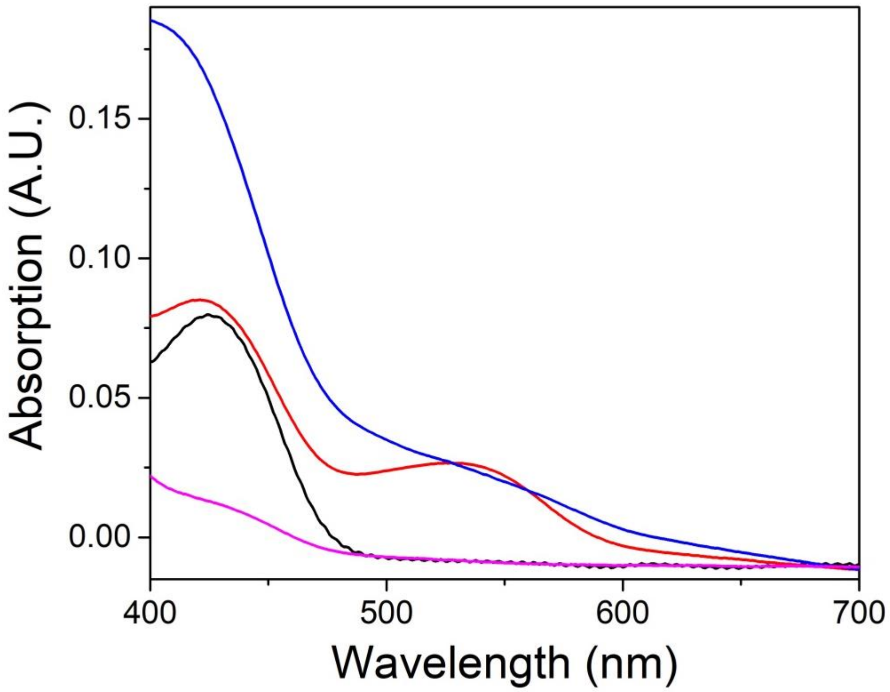

Supplementary Figure 5: UV-Vis absorption spectra of 1,3-bis(2,4-dinitrophenyl)-imidazolium bromide in acetonitrile before (black-colored spectrum) and following addition of KO^{t}Bu (red-colored spectrum). UV-Vis absorption spectra of 1,3-bis(2,4-dinitrophenyl)-imidazolium bromide in acetonitrile while applying a voltage of -1 V on Au electrode in the absence of water (magenta-colored spectrum) and with 50 mM H_2O (blue-colored spectrum).

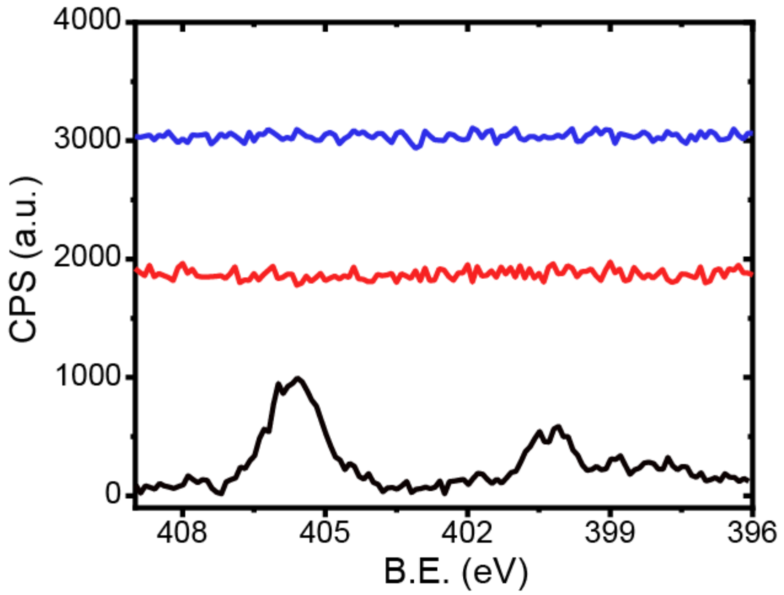

Supplementary Figure 6: N1*s* XPS spectra of Au electrode following its exposure to 5 mM of 1,3-bis(2,4-dinitrophenyl)-imidazolium bromide in acetonitrile with 50 mM water and while applying -0.5 V (blue-colored spectrum); applying -1 V without water addition (red-colored spectrum); and while applying -1 V with 50 mM of water (black-colored spectrum).

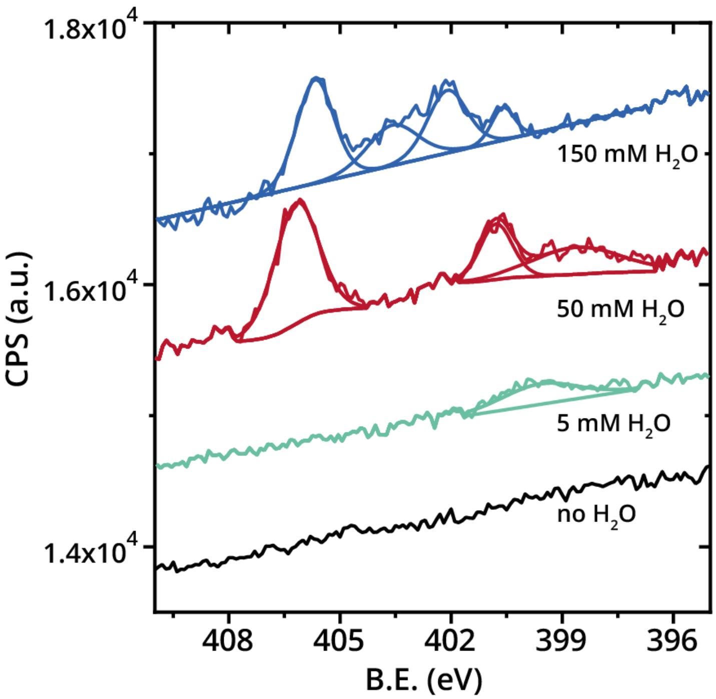

Supplementary Figure 7: N1*s* XPS spectra of EC-deposited NO_2-NHCS following EC deposition with various concentration of H_2O in the solution. EC deposition conditions: 5 mM of 1,3-bis(2,4-dinitrophenyl)-imidazolium bromide in acetonitrile and applying -1 V.

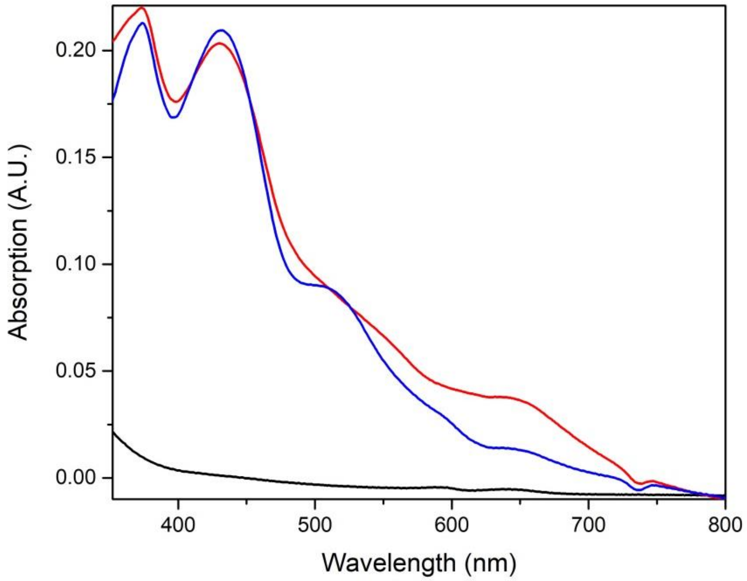

Supplementary Figure 8: UV-Vis absorption spectra of 1,3-bis(2,4-dinitrophenyl)-imidazolium bromide (10 µM) in DMSO before (black-colored spectrum) and following addition of 10 µM KO^tBu (red-colored spectrum) and KOH (blue colored spectrum).

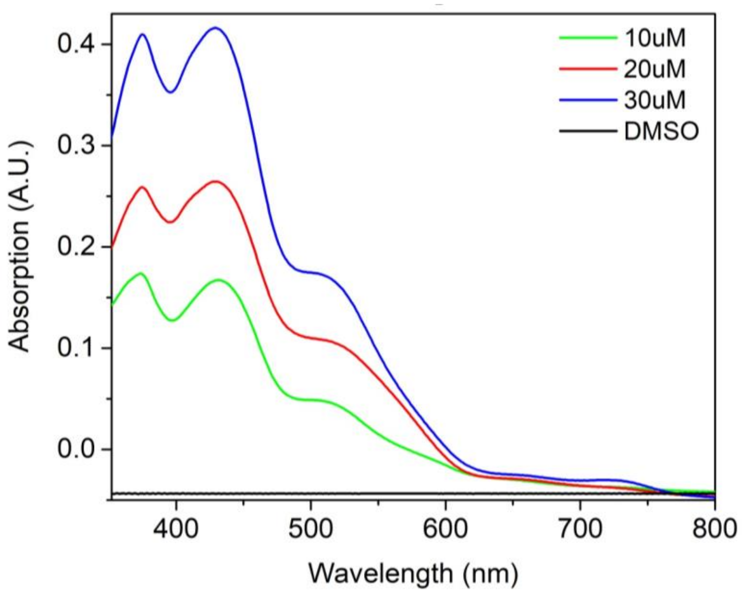

Supplementary Figure 9: UV-Vis absorption spectra of 10, 20 and 30 μM 1,3-bis(2,4-dinitrophenyl)-imidazolium bromide in DMSO following addition of 0.01 M KOH (green-, red- and blue-colored spectra, respectively).

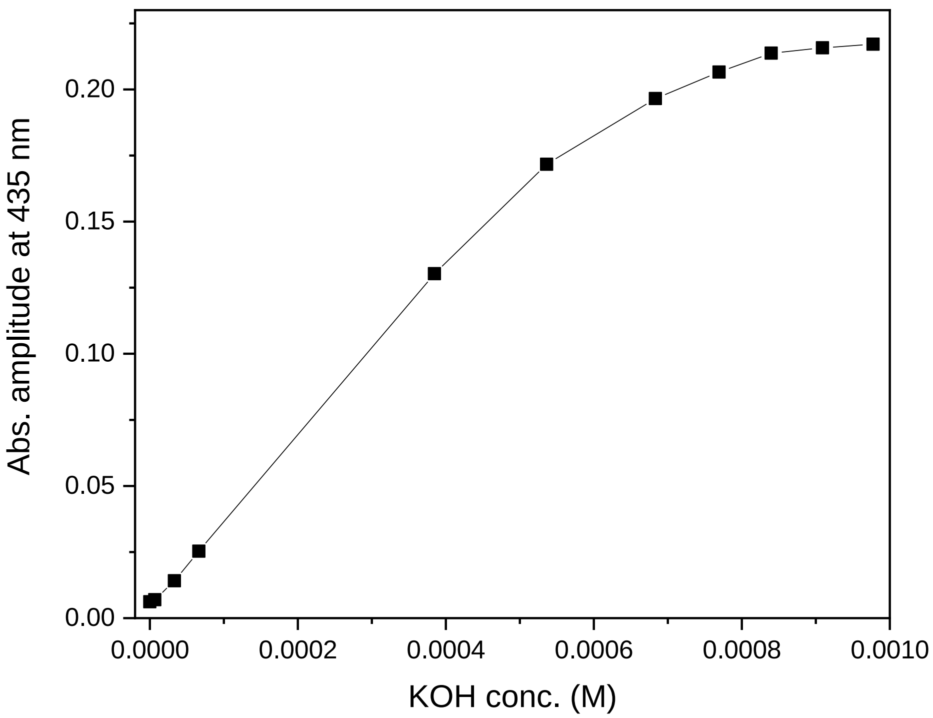

Supplementary Figure 10: Amplitude of the absorption peak at 435 nm as function of KOH concentration with 1,3-bis(2,4-dinitrophenyl)-imidazolium bromide (10µM) in DMSO .

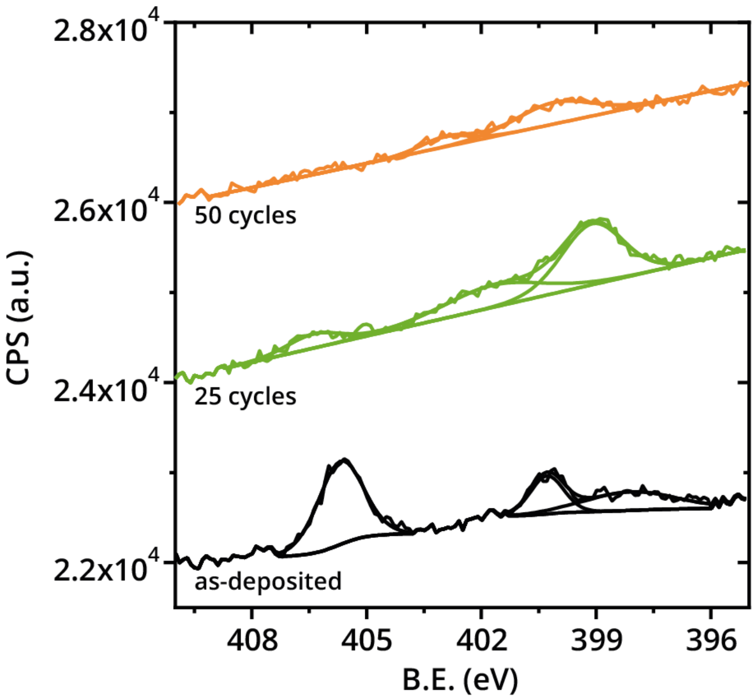

Supplementary Figure 11: N1*s* XPS spectra of Au electrodes following EC-deposition of NO_2-NHCs (black-colored spectrum), and after 25 and 50 cyclic voltammograms (CV) cycles (green and orange colored spectra, respectively). CV conditions: 0.1 M KNO_3 aqueous solution, reference electrode - Hg/Hg_2SO_4, counter electrode - Pt, scan range −0.5 V to 1 V, scan rate 0.1 V/sec.

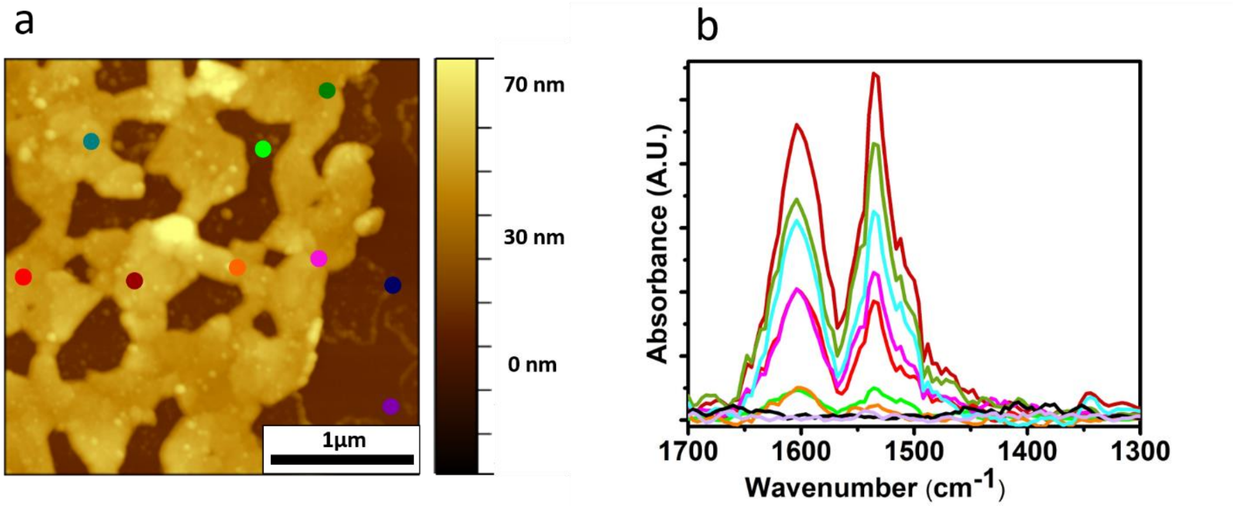

Supplementary Figure 12: AFM topography (**a**) and AFM-IR point spectra measurements (**b**) of EC-deposited NO_2-NHCs. Colored circles in **a** mark the local IR measurement positions and the measured IR spectra are shown in **b** with identical color-coding.

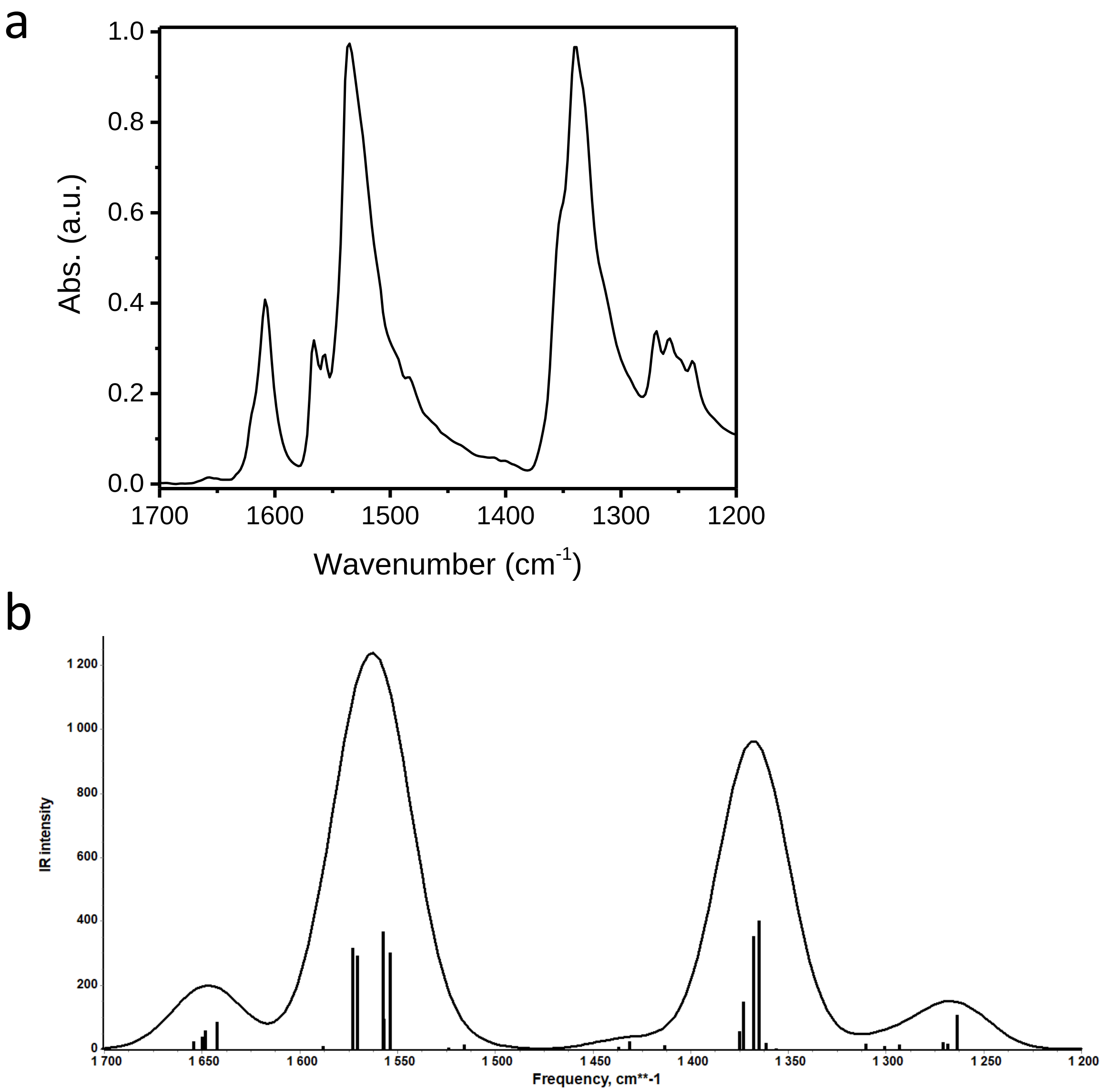

Supplementary Figure 13: ATR-IR spectrum (**a**) and calculated IR spectrum (**b**) of 1,3-bis(2,4-dinitrophenyl)-imidazolium bromide.

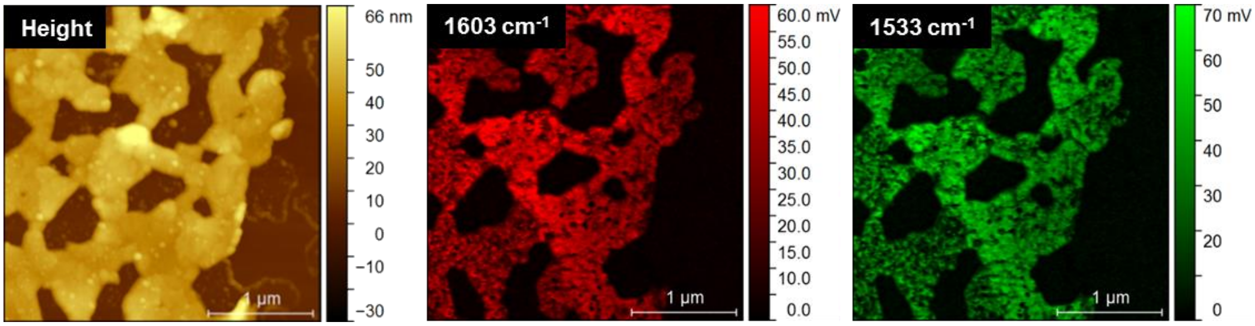

Supplementary Figure 14: AFM topography and AFM-IR mapping at 1603 and 1533 cm^{-1} of EC-deposited NO_2-NHCs.

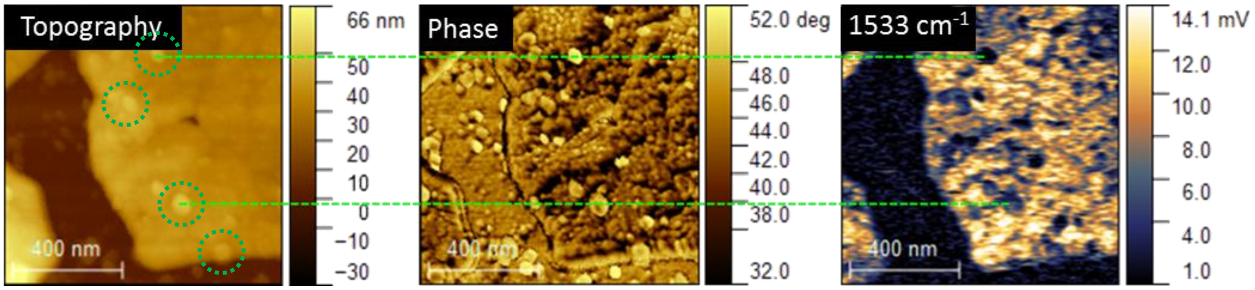

Supplementary Figure 15: AFM topography, AFM phase imaging and AFM-IR mapping (at 1533 cm^{-1}) of EC-deposited NHCs. Green circles highlight the appearance of randomly distributed structures in the AFM topography image and green lines connect the appearance of these structures in the three images.

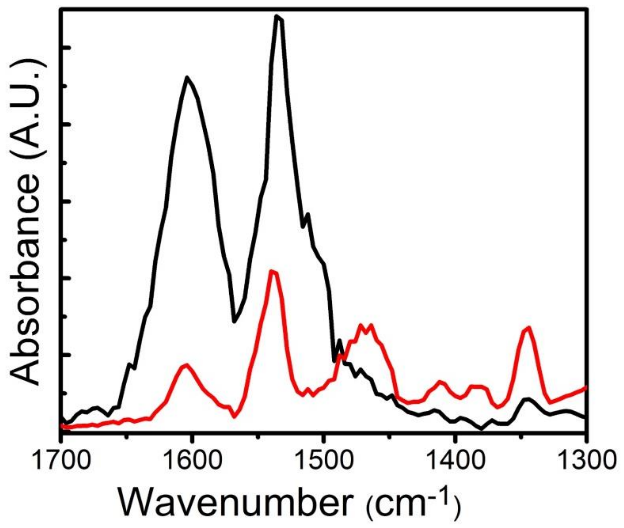

Supplementary Figure 16: AFM-IR spectra of EC-deposited and base-induced deprotonation deposited NO_2-NHC on Au film (black and red colored spectra, respectively).

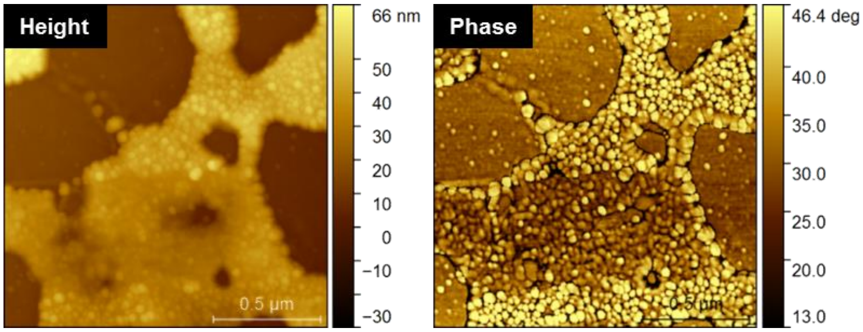

Supplementary Figure 17: AFM topography and AFM phase image of NO_2-NHCs that were deposited by base-induced deprotonation.

Supplementary Tables

Supplementary Table 1: XPS analysis of NO_2-NHCs monolayer formation by EC-deposition and base-induced deprotonation

	NO_2/NH_x [a]	N1*s*/Au4*f* [b]	N:Br:K [c]
EC-deposition	1.5	$13.4 \cdot 10^{-3}$	1:0.3:0
Base-induced deprotonation	0.07	$5 \cdot 10^{-3}$	1:1.25:0.4

[a] Ratio was quantified by analysis of the high and low energy peaks area in the N1*s* XPS spectra. [b] Ratio was quantified by analysis of the peaks area ratio. [c] Atomic ratio was analyzed based on XPS data and normalized to the ionization cross section of each element.

Supplementary Table 2: IR signals for imidazolium salt precursor and surface-anchored NO_2-NHC

Molecule	Imidazolium salt precursor		NO_2-NHC (EC deposition)	NO_2-NHC (Base-activated)	NO_2-NHC (Base-activated) [a]
Method	ATR-IR	DFT	AFM-IR	AFM-IR	IRRAS
Symmetric N=O	1340 cm^{-1}	1368 cm^{-1}	-	1346 cm^{-1}	1347 cm^{-1}
N-H	-	-	-	1466 cm^{-1}	-
Asymmetric N=O	1536 cm^{-1}	1562 cm^{-1}	1536 cm^{-1}	1533 cm^{-1}	1544 cm^{-1}
C=C	1608 cm^{-1}	1645 cm^{-1}	1608 cm^{-1}	1603 cm^{-1}	1615 cm^{-1}

[a] Values extracted from Chem. Eur. J. 25, *2019*, 15067

Supplementary Table 3: XPS analysis of NO_2-NHCs monolayer on various metals

	NO_2/NH_x [a]	N1*s*/Metal [b]	N:Br:K [c]
Pt	0.064	0.024	1:0:0
Au	1.5	0.013	1:0.3:0
Pd	0.080	0.008	1:1.2:0
Ag	0.006	0.009	1:0.7:0

[a] Ratio was quantified by analysis of the high and low energy peaks area in the N1s XPS spectra. [b] Ratio was quantified by analysis of the peaks area ratio. [c] Atomic ratio was analyzed based on XPS data and normalized to the ionization cross section of each element.

Supplementary Methods

DFT calculations

In order to determine the area of NO_2-NHC on Au (111) substrate, we performed ionic relaxations using Density Functional Theory (DFT) with two types of calculations:

1. Single NHC-NO_2 molecule: Modelled using a large unit-cell in the DFT calculations with Au (111) substrate with 9x9 atoms in the horizontal plane. The NO_2-NHC molecules were spaced 26 Å apart to ensure a minimum separation of 12 Å between atoms from different molecules, and guaranteed that the molecules did not interact.

2. Closely-packed NHC-NO_2 molecules: Modelled using a small unit-cell containing two NO_2-NHC molecules and Au (111) substrate with 6x6 atoms in the horizontal plane. We considered the maximally closed-packed configuration in which the nitro-aryl groups are parallel to the surface. In this configuration the closest molecules were 7.7 Å apart and the closest intermolecular atomic distances were 1.2 Å (O-O), 1.8 Å (O-H) and 1.8 Å (H-H) before relaxation. After relaxation the intermolecular distances increased to 3.1 Å (O-O), 2.1 Å (O-H) and 2.5 Å (H-H) in order to increase the distance of the repulsive O-O and H-H interactions while minimizing the distance of the attractive O-H interaction. The -NO_2 groups rotated in order to maximize the distance between neighboring intermolecular oxygen atoms. For comparison we also performed the calculation for lone NHC-NO_2 molecules in the 6x6 cell (nearest intermolecular atomic distances are 3.3 Å (between intermolecular oxygen atoms). We found that the binding energy per molecule without dispersive forces was identical for the lone NHC-NO_2 molecule and the closely-packed NHC-NO_2 molecules. When the relaxation was performed with dispersive forces the closely packed molecules were characterized with higher binding energy per molecule of 0.1 eV due to the attractive O-H bonds.

All calculations were performed using the VASP[1] software, with PAW[2] pseudopotentials, and the PBE[3] exchange-correlation functional. Results were converged to an accuracy of approximately 0.03 eV, in relation to the cutoff energy for the planewave basis (converged at 400 eV), the vacuum length (set at 20 Å above the Au (111) surface), and the k-point mesh density. The Au (111) substrate consisted of 4 layers of Au atoms. The structures were relaxed using ionic relaxation with the conjugate gradient algorithm, with all atoms free to move except the bottom two Au layers which were kept fixed with the interatomic distance determined from minimizing the energy of bulk Au. Van der Waals dispersion corrections were calculated using the Tkatchenko-Scheffler[4] method.

For a unit cell with n NHC-NO_2 molecules, we calculated the binding energy per unit cell of the NHC-NO_2 molecules by comparing the relaxed energy of the NHC-NO_2 molecule-Au (111)

surface complex with the energy of the isolated Au (111) relaxed surface plus the energy of *n* isolated relaxed molecules. The binding energy per molecule was calculated by dividing the binding energy per unit cell by *n*. The surface area of the relaxed lone NHC-NO_2 molecule on the Au (111) surface was approximately 15 Å×7 Å =105 $Å^2$. The surface area of closely packed NHC-NO_2 molecules was 130 $Å^2$ per molecule.

Gas-phase calculations of the structure and IR spectra of the imidazolium salt precursor were calculated using DFT. The optimal structure was optimized using the PBE functional[3] with a cc-pVTZ basis set.[6] During the optimization in order to converge to a state where the negative charge is on the Br we partitioned the system into fragments using the constrained locally-projected SCF for molecular interactions (SCF-MI) procedure.[7] The IR spectrum was then calculated within the harmonic approximation using M06-2X functional[8] with a cc-pVTZ basis set. Gaussian broadening was applied.

ATR-IR

Attenuated total reflectance Fourier transform infrared (ATR-FTIR) measurements of 1,3-bis(2,4-dinitrophenyl)-imidazolium bromide were conducted using a Thermo Scientific Nicolet iS50 instrument and a diamond ATR crystal.

pK_a measurements of 2,4 dinitrophenyl-imidazolium and pH analysis in the vicinity of the electrode

The pK_a of 1,3-bis(2,4-dinitrophenyl)-imidazolium was calculated by titration with KOH in DMSO while monitoring the changes in the carbene concentration by UV-Vis absorption spectroscopy measurements. Supplementary Figure 8 shows the absorption spectra of imidazolium salt in DMSO before and after addition of KOH and KO^tBu, demonstrating the similarities in the carbene absorption pattern following deprotonation by the two bases. KOH was used as a base for the titration experiments due to its simple 1:1 stoichiometric ratio in acid-base reaction with the imidazolium. The influence of KOH addition on the UV-Vis absorption spectra of the imidazolium salt solution in DMSO is shown in Supplementary Figure 9. Analysis of the peak amplitude at 435 nm as function of the KOH concentration is shown in Supplementary Figure 10.

pK_a analysis was performed by determining, based on UV-Vis absorption spectra, the amount of KOH that was needed in order to deprotonate half of the imidazolium salt, using the following equation:

$$[imidazolium]^{+} + [OH]^{-} \leftrightarrow [carbene] + [H_2O]$$

$$K = \frac{[imidazolium]^{+}[OH]^{-}}{[carbene]}$$

At half equivalent point of the titration ($A_{1/2}$=A_{max}/2) the concentrations of carbene and salt are equal and therefore the pK_a can be directly analyzed. Using this method the pK_a was calculated for three different initial concentrations of imidazolium salt (10, 20 and 30 μM), yielding:

pK_a = 10.49 ± 0.02

The pH on the electrode surface was calculated based upon the following:

Area of the electrode (A) = 0.35 cm^2

The distance that the hydroxide ions travel from the electrode depends on their diffusion coefficient (D_{OH}) and the deposition duration (assuming linear diffusion from a rectangular electrode).

D_{OH} = 5.3E-5 [cm^2/sec] (diffusion in water)

The electrodeposition duration (t) was 5 min (300 sec):

Distance = sqrt (D_{OH} · t) = sqrt (5.3E-5 cm^2/sec · 300 sec) = 0.126 cm

The volume of the diffusion layer (V_{OH}) equals the area of the electrode (A) multiplied by the linear diffusion distance of the ions:

V_{OH} = A · Distance = 0.35 cm^2 · 0.126 cm = 0.044 mL

Water reduction reaction: e^{-} + H_2O → 0.5H_2 + OH^{-}

Q (total charge in coulomb) = I · t = 0.5 mA · 300 sec = 0. 15 C

Dividing in Faraday's constant will yield the number of moles: N = Q / F = 0. 15 C / 96,485 C/mol = 1.55 E-6 mol

Now, the concentration of OH^{-} can be calculated: C_{OH^-} = N/V = 1.55E-6 mol / 0.044 mL = 3.53 E-2 [M]

C_{H3O+} = 10^{-14} / 3.53 E-2 M = 2.83 E-13 M

pH = -log(C_{H3O+}) = 12.54

Quantitative analysis of surface coverage by NO_2 electroreduction:

Electrochemical deposition

Total charge during electroreduction = 1.87E-6 [A·V] / 0.1 [V·sec^{-1}] = 1.87E-5 [C] (total charge)

1.87E-5 [C] / 24 electrons = 7.8E-7 [C] (total charge normalized to # of molecules)

7.8E-7 [C] / 96485 [C·mol^{-1}] = 8.07E-12 [mol]

8.07E-12 [mol] / 0.35 [cm^{2}] = 2.3 E-11 [mol/cm^{2}] (Surface coverage)

Base-induced deprotonation deposition

Total charge during electroreduction = 3.11E-7 [A·V] / 0.1 [V·sec^{-1}] = 3.11E-6 [C] (total charge)

3.11E-6 [C] / 24 electrons = 1.295E-7 [C] (total charge normalized to # of molecules)

1.295E-7 [C] / 96485 [C·mol^{-1}] = 1.343E-12 [mol]

1.343E-12 [mol] / 0.35 [cm^{2}] = 3.83 E-12 [mol/cm^{2}] (Surface coverage)

Supplementary References

[1] G. Kresse; J. Furthmüller, Phys. Rev. B 54, 11169 (1996).

[2] a. P. E. Blochl. Phys. Rev. B, 50, 17953 (1994) b. G. Kresse; D. Joubert, Phys. Rev. 59, 1758 (1999).

[3] J. P. Perdew; K. Burke; M. Ernzerhof, Phys. Rev. Lett. 77, 3865 (1996).

[4] A. Tkatchenko; M. Scheffler, Phys. Rev. Lett. 102, 073005 (2009).

[5] Y. Shao, et al. Molecular Physics 113, 184 (2015).

[6] T. H. Dunning Jr, J. Chem. Phys. 90, 1007 (1989).

[7] N. Mardirossian; M. Head-Gordon, Mol. Phys. 115, 2315 (2017).

[8] Y. Zhao; D. G. Truhlar, Theor. Chem. Acc. 120, 215 (2006).
